# Diagnostics of Articular Cartilage Damage Based on Generated Acoustic Signals Using ANN—Part II: Patellofemoral Joint

**DOI:** 10.3390/s22103765

**Published:** 2022-05-15

**Authors:** Robert Karpiński, Przemysław Krakowski, Józef Jonak, Anna Machrowska, Marcin Maciejewski, Adam Nogalski

**Affiliations:** 1 Department of Machine Design and Mechatronics, Faculty of Mechanical Engineering, Lublin University of Technology, Nadbystrzycka 36, 20-618 Lublin, Poland; j.jonak@pollub.pl (J.J.); a.machrowska@pollub.pl (A.M.); 2 Department of Trauma Surgery and Emergency Medicine, Medical University of Lublin, Staszica 11, 20-081 Lublin, Poland; adam.nogalski@umlub.pl; 3 Orthopaedic Department, Łęczna Hospital, Krasnystawska 52, 21-010 Łęczna, Poland; 4 Department of Electronics and Information Technology, Faculty of Electrical Engineering and Computer Science, Lublin University of Technology, Nadbystrzycka 36, 20-618 Lublin, Poland; m.maciejewski@pollub.pl

**Keywords:** vibroacoustic signal, osteoarthritis, patellofemoral joint, kinetic chain, artificial neural networks, multilayer perceptron

## Abstract

Cartilage loss due to osteoarthritis (OA) in the patellofemoral joint provokes pain, stiffness, and restriction of joint motion, which strongly reduces quality of life. Early diagnosis is essential for prolonging painless joint function. Vibroarthrography (VAG) has been proposed in the literature as a safe, noninvasive, and reproducible tool for cartilage evaluation. Until now, however, there have been no strict protocols for VAG acquisition especially in regard to differences between the patellofemoral and tibiofemoral joints. The purpose of this study was to evaluate the proposed examination and acquisition protocol for the patellofemoral joint, as well as to determine the optimal examination protocol to obtain the best diagnostic results. Thirty-four patients scheduled for knee surgery due to cartilage lesions were enrolled in the study and compared with 33 healthy individuals in the control group. VAG acquisition was performed prior to surgery, and cartilage status was evaluated during the surgery as a reference point. Both closed (CKC) and open (OKC) kinetic chains were assessed during VAG. The selection of the optimal signal measures was performed using a neighborhood component analysis (NCA) algorithm. The classification was performed using multilayer perceptron (MLP) and radial basis function (RBF) neural networks. The classification using artificial neural networks was performed for three variants: I. open kinetic chain, II. closed kinetic chain, and III. open and closed kinetic chain. The highest diagnostic accuracy was obtained for variants I and II for the RBF 9-35-2 and MLP 10-16-2 networks, respectively, achieving a classification accuracy of 98.53, a sensitivity of 0.958, and a specificity of 1. For variant III, a diagnostic accuracy of 97.79 was obtained with a sensitivity and specificity of 0.978 for MLP 8-3-2. This indicates a possible simplification of the examination protocol to single kinetic chain analyses.

## 1. Introduction

The knee joint consists of two separate but connected joints, namely the tibiofemoral and patellofemoral joints (PFJ). Both joints work in conjunction and enable the knee joint to play vital role in walking, which is crucial for daily activities. The patella and patellar groove of the femur are covered with typical hyaline cartilage. Hyaline cartilage is a highly specialized tissue devoid of nerve endings and vessels. During daily activities, the cartilage in the patellofemoral joint is subjected to high loads, especially during kneeling, squatting, and ascending and descending stairs [1]. The forces on the joint are variable and depend on the degree of knee flexion and whether the foot is in contact with the ground [2]. Chondrocytes account for 1–5% of dry cartilage mass [3]. Due to the sparse distribution of chondrocytes and the lack of vessels, cartilage has a very limited healing potential [4]. Moreover, the superficial cartilage layer is responsible for withstanding shearing forces, which act on the cartilage during daily activities. Injury to the superficial layer leads to the irreversible cartilage derangement of deeper cartilage layers [5]. Wear of the hyaline cartilage can be caused by multiple factors including obesity, repetitive deep knee flexion, or malalignment of the PFJ [6]. The central part of the patella has the thickest cartilage layer in the human body [7], which is a result of the continuous loads exerted during daily activities. The loads in the PFJ increase with the knee flexion angle [8]. This continuous overload can easily lead to the disruption of the superficial and deeper layers of cartilage, which inevitably leads to the development of osteoarthritis (OA) [9]. The main symptoms of OA are pain and the limitation of the function of the affected joint. Until now, no treatment to restore damaged hyaline cartilage has been proposed. The prevalence of PFJ joint space narrowing is radiologically apparent in 33.7% of men and 26.1% of women >60 years of age [10].

Various methods of treatment, including conservative and surgical measures have been proposed for the treatment of OA [11,12,13,14]. The end stage disease is indicated for arthroplasty. Even though arthroplasty is considered as a gold standard for the end stage disease, after the 24-month followup, nearly 20% of patients were not satisfied with the procedure [15]. Therefore, researchers seek other treatment options, especially joint preserving and biological treatment in order to delay the need for arthroplasty as long as possible. To achieve that, OA has to be diagnosed at the early stages, so that adequate treatment can be implemented. Diagnosis of OA is usually established on conventional X-ray with the use of the Kellgren-Lawrence 0–4 scale [16]. This scale reflects adaptive changes in the subchondral bone presenting as osteophytes and joint space narrowing [17]. Therefore, such evaluation is adequate for monitoring the disease but not suitable for early detection of chondral lesions. An ultrasound has also been proposed as an imaging modality to evaluate cartilage status; however, the ability to detect early chondral degeneration does not differ significantly from a conventional X-ray [18]. Magnetic resonance imaging (MRI) is the imaging modality used most frequently for detection of chondral lesions. Over the decades, the availability of MRI has increased; nevertheless, the waiting time is still long, and the examination is expensive, requiring dedicated radiologists for proper image evaluation. Moreover, the published sensitivity of detection of chondral lesions differ from 45% to 94% between authors [19,20]. Our own research has shown that MRI grossly underestimates the chondral lesion grade, and diagnostic accuracy increases with the increase in chondral lesion grade [21,22]. MRI became the modality of choice among orthopedic surgeons and general practitioners without recognition of its deficiencies. Up to 20% of patients referred for a knee MRI have not had a previous physical examination [23]. Moreover, some authors show that an MRI acquired before planned arthroscopic intervention does not change the course of treatment and has little impact on overall costs of the procedure [24].

The development of diagnostic tools providing an alternative to classical imaging diagnostic methods, enabling inexpensive, noninvasive, and precise diagnosis of osteoarthritis even at an early stage seems to be extremely important in clinical practice. Automatic diagnosis of diseases using machine learning methods is now widely used in medicine [25,26,27,28,29]. Machine learning is most commonly is utilized in radiology for object detection, segmentation, and classification [30] A growing interest can also be observed in nuclear medicine, where machine learning is utilized, e.g., for the glomerular filtration rate in SPECT/CT [31]. Neurology is another field in which machine learning is gaining recognition for the early diagnosis of Alzheimer disease [32]. In orthopedics, ANNs are proposed as methods for risk calculation in patients with hip fractures [33]. As shown, machine learning methods have gained interest throughout the medical field, and the use of numerical methods may prove to also be a valuable tool to extend the methods used to assess damage to joint structures. In particular, machine learning and deep learning methods may prove useful especially in cases that require solving classification, detection, and related problems without the involvement of a radiologist [34,35,36].

During normal movements of the knee joint, both intra-articular and extra-articular structures can produce vibrations or sounds as they move relative to each other [37,38]. The sound generated by the knee joint during movement (flexion or extension) is called a vibroarthrographic (VAG) signal [38,39,40]. Changes in the mechanical properties and structure of the articular cartilage surface, such as the appearance of bumps, cracks, or cartilage defects in the successive stages of degenerative changes affect the vibroacoustic signals recorded during movement of the knee joint [41,42,43]. These signals are generated by transient elastic waves resulting from sudden stress redistribution in the material and can be recorded from the surface of the knee [44]. In 1902, Blodgett [45] published his paper on the correlation between the sounds generated by the knee joint on auscultation and OA. Throughout the 20th and 21st centuries, numerous groups of researchers have developed this method of assessing articular cartilage from both acoustic [46,47,48,49,50,51,52] and vibrational signals [53,54,55,56,57,58]. Vibroarthrography (VAG), which is a measurement of the vibrations or sounds generated during joint movement, has achieved >90% accuracy in detection OA in the knee joint [59]. Despite many years of work, there are no clear criteria for using vibroacoustic diagnostics in everyday clinical practice. Moreover, according to the literature, there is a wide discrepancy among researchers in both classification methods and the obtained results. Diagnostic accuracy in different studies ranges from 68.9% [60] to 100% [61]. Even greater differences are found in sensitivity, which in the case of screening modality is of paramount value. Depending on the classification methods, the same authors obtained sensitivity ranging from 0.711 [55] to 1 [61]. Such differences in obtained results require further studies to find the best classification methods and examination protocol, which could then be implemented in clinical practice.

The purpose of this study is to evaluate the usefulness of acoustic signals generated by the patellofemoral joint during motion for sequences recorded in an open and closed kinetic chain as a potential tool for the diagnosis of osteoarthritis. The aim is also to select an optimal testing protocol that will provide the best diagnostic results while simplifying the testing procedure. For this purpose, an attempt was made to use ANN to classify the cases based on selected measures of acoustic signals treated as potential indicators of the state of articular cartilage damage in different variants of kinetic chains. MLP and RBF type networks were used for classification. A significant contribution is also the fact that the study, in addition to a typical physical examination and diagnostic imaging, includes a precise intraoperative assessment of the degree and location of cartilage damage, which has not been used in previous studies. This work extends the authors’ previous work with data for new anatomical locations (patella) and provides information on the utility of the proposed testing protocol. In the future, this may enable the development of a diagnostic method that allows for precise diagnosis of the degree of cartilage damage, taking into account its location and allowing for the diagnosis of other typical injuries to structures within the knee joint such as ligaments or meniscus.

## 2. Materials and Methods

Vibroacoustic assessment, referred to as vibroarthrography for joint diagnosis, may be an alternative to the classic diagnostic methods used in the evaluation of typical injuries to knee joint structures [62,63]. This method is based on the analysis of vibroacoustic signal. It is often used in machine diagnostics due to the fact that it is a nondestructive method allowing for continuous monitoring of a moving object. A normal knee joint with a smooth and well-lubricated cartilage surface should move fluently and quietly, whereas a diseased joint with a “rough” and poorly-lubricated cartilage surface may move unevenly, producing vibrations and acoustic signals [48]. Analysis of the vibrations and sounds generated by a moving knee joint can help to better understand the associated pathological conditions [64] and, ultimately, provide a valuable diagnostic tool. However, due to the lack of clear guidelines and developed testing protocols, as well as the lack of dedicated diagnostic equipment, this method has not yet been widely used in clinical practice.

### 2.1. Participants

In total, 67 patients were included in this study. The study group consisted of 15 males and 19 females, and the control group consisted of 9 males and 24 females. In the control and study groups, the mean age was 24.10 (years) and 56.15 (years) respectively. Detailed participant group characteristics are shown in Table 1. All patients and healthy volunteers signed written consent prior to participation in the study. Identical questionnaires were filled out by both groups, and identical physical examination was performed. In the control group, signals recorded for both knee joints (66 total) were analyzed, while in the study group, only the operated knee joint (34 total) was taken into account. The physical examination in study group was performed one day prior to surgery, and VAG acquisition was performed immediately after physical examination in both groups. All patients in the study group were qualified for surgical treatment based on their medical history, physical examination, and radiological findings, after detailed evaluation performed by a specialized orthopedic surgeon. The study group consisted of two subgroups, patients qualified for arthroscopic treatment and patients qualified for total knee replacement. All patients in the TKR (Total Knee Replacement) subgroup showed significant chondral lesions in the knee joint, whereas in the arthroscopic subgroup, several patients showed no chondral lesions and were treated for other intraarticular lesions such as meniscal or ligamentous tears. Exclusion criteria from the control group included: any previous history of trauma or joint disease and any positive finding on physical examination prior to VAG acquisition. The study received approval from the Bioethics Committee of the Medical University of Lublin consent number KE-0254/261/2019.

### 2.2. Physical Examination

Medical history was collected from all patients and healthy participants. Limb alignment and any signs of joint swelling or pain on palpation in both groups was noted. Passive as well as active range of motion (ROM) were evaluated in each group. Following that, special tests were introduced such as the McMurray, Apley, and Thessaly [65,66,67] for evaluation of meniscal lesions. Varus and valgus stress was applied to the knee joint in order to detect any collateral ligament instability. The anterior cruciate ligament was evaluated with the use of a lever sign, pivots shift, the Lachman test, and the anterior drawer test [68,69,70,71]. For evaluation of the patellofemoral joint (PFJ), palpation of the PFJ, patella shifting, and the patellar grind test were introduced [72,73]. Based on the physical examination, initial classification into the OA group was made for findings suggestive of damage to knee joint structures. Any positive physical examination findings, on the other hand, were treated as exclusion criteria for the HC group.

### 2.3. Surgical Treatment

**Figure 1:**
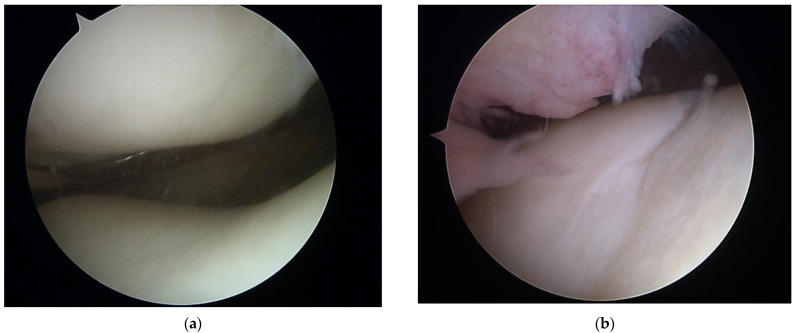
Arthroscopic view of patellofemoral joint with healthy cartilage (**a**) and grade III chondral lesions in the patellar groove of the femur (**b**).

**Figure 2:**
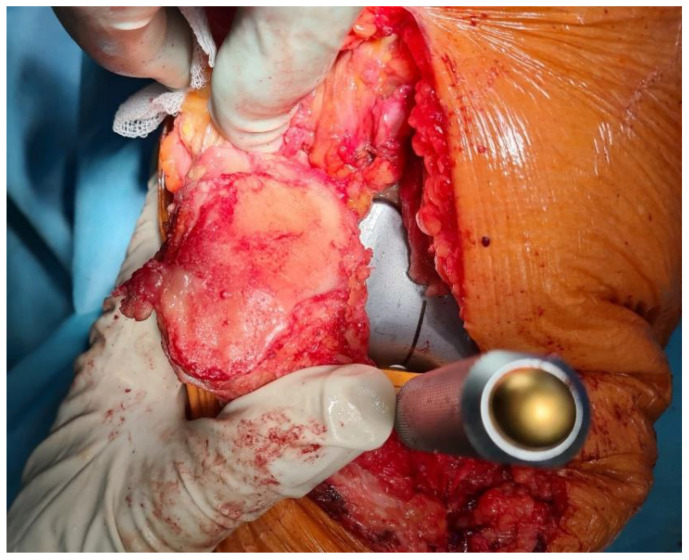
Luxation of patella for evaluation of patellar cartilage during trial placement of TKR components.

Both arthroscopic and TKR surgeries were performed in a typical manner by orthopedic surgeons trained and specialized in this field. For arthroscopy, a typical 30-degree scope was utilized and standard anteromedial and anterolateral portals created for instrument placement. After introduction of the scope into the joint cavity, a standardized knee evaluation was performed with the probing of cartilage. The degree of cartilage damage was evaluated according to the International Cartilage Repair Society (ICRS) guidelines [74,75]. The same classification was used for evaluation of chondral lesions during TKR; however, in those surgeries evaluation was performed without magnification of the arthroscope. In this study, we have not included any TKR with patellar resurfacing; therefore, the patellar cartilage was evaluated in situ, and the patellar groove of the femur was evaluated after resection of the articular surface. This approach was based on a surgical technique that was not altered for this study. Arthroscopic and direct visualization of the cartilage provide the best evaluation method, which was shown in exemplary views from an arthroscopic evaluation of a PFJ in Figure 1 and direct visualization during TKR in Figure 2.

### 2.4. Signal Acquisition

Data acquisition required construction of a proper measuring system dedicated to the task. The proposed solution included the following components:

- Orthesis with a rotary encoder and vibration transducer;
- Microcontroller with peripherals for signal acquisition;
- Computer with data recording software.

**Figure 3:**
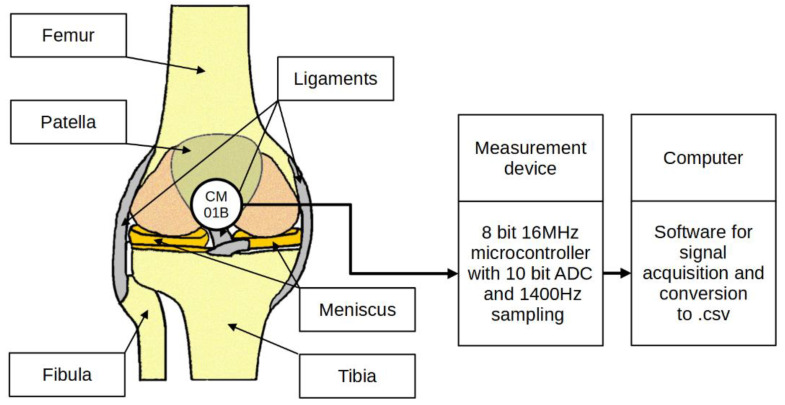
Placement of the sensor and measurement concept.

**Figure 4:**
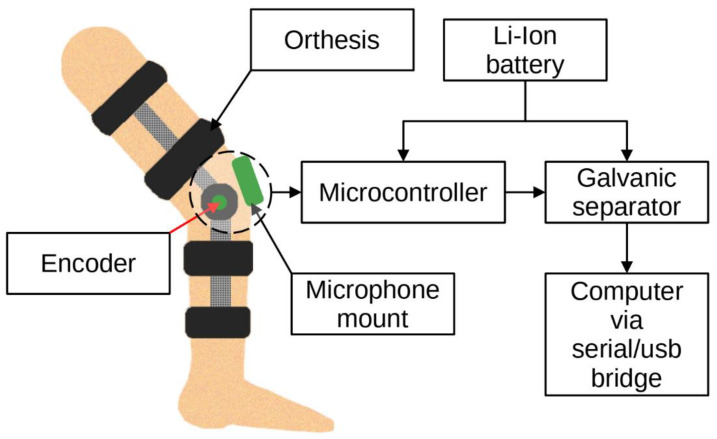
Idea of the measurement system.

Bone vibrations were converted to electrical signals by a dedicated CM01B solid body microphone [76] placed on the patella as shown in Figure 3. This device allowed for conversions of signals in the 10 Hz to 2 kHz bandwidth. Knee rotation was measured using a rotary encoder from Bourns [77], which provided a 10-bit continuous digital output based on rotation of an embedded magnet in 360 degrees, ten times per second. Additional hard stops were placed on the orthesis joint, limiting the rotation between 0 and 90 degrees. The analog data from the microphone were sampled by an Atmega2560 8-bit microcontroller with an analog to digital converter with 10 bits of precision. The sampling rate used was 1400 Hz with 10-bit resolution. Acoustic signals were recorded during lower limb movement in an open and closed kinetic chain. A kinetic chain is an engineering concept used to describe human movement that finds widespread use in clinical applications including sports medicine, rehabilitation, prosthetics, and orthotics [78]. We can define an open kinetic chain as a combination of sequentially arranged joints in which the distal segments are free to move, an example being the movement of the knee in a sitting position. The movements of the individual segments are independent of each other in this case. A closed kinetic chain is a system in which the distal segment is not free to move. The motion of one segment causes a specific motion of the other segments, and each segment is connected to at least two other segments [78]. For both closed and open kinetic chain tests, the time of one cycle involving 90°–0°–90° movement was approximately 2 s. Signals were recorded for 10 complete repetitions of the described procedure. Obtaining a uniform length of the recorded signals was not possible due to the variability resulting from the individual limitations of the test subjects. The stream of vibration and rotation data was sent via a serial/usb interface. Patient safety was ensured by means of galvanic isolation using an ADuM4160 USB 2.0 isolator chip [79]. The power source for the device was an 11.1 V 3 s Li-Ion battery. The main modules of the system are shown in Figure 4. Data were recorded by a computer and saved as a comma-separated values file. Both open and closed kinematic chains were used during measurements.

**Figure 5:**
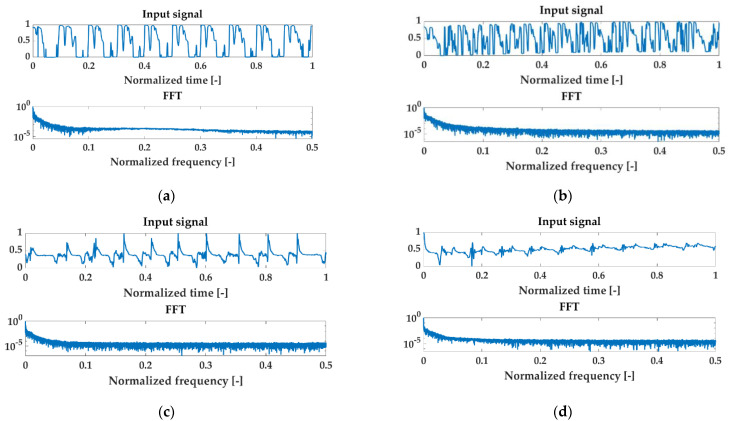
Examples of normalized signals for healthy (HC) and injured knees (OA) in the time and frequency domain for open kinetic chain (OKC) and closed kinetic chain (CKC) recorded with a sensor patella. Respectively: (**a**) HC OKC, (**b**) OA OKC, (**c**) HC CKC, and (**d**) OA CKC.

Examples of normalized signals for healthy and injured knees in the time and frequency domain for OKC and CKC recorded with a sensor placed on the patella are shown in Figure 5.

### 2.5. Signal Preprocessing

VAG waveforms are not free from certain disturbances and artifacts such as data series before and after movements, random noise, or electrical network interference. Before starting the data analysis, it was necessary to reduce these, so they did not affect the obtained results. In the first stage, the initial signal preparation procedure involved cutting the signal in the regime of the limb movement cycles. It was carried out on the basis of the signal from the encoder, indicating the start and end of the movements. The raw signal was subjected to the reduction in irrelevant time series using the automatic slope detection procedure, which detects the beginning and end of the work cycles in the encoder signal. At the second stage of signal preparation, frequency filtering was performed using the EEMD (Ensemble Empirical Mode Decomposition) algorithm. This tool is widely used in cleaning biomedical signals [80,81,82,83], including those directly related to VAG analyses [58]. It is related to the high efficiency of artifact reduction in the form of hard to avoid random noise.

The modal decomposition procedure was introduced by Huang [84], which has become an effective tool for filtering and analyzing nonlinear and nonstationary signals, a challenge for researchers. It consists in distinguishing a series of time waveforms called Intrinsick Mode Functions (IMFs) grouped in the area of specific frequency values. The discussed algorithm consists of several stages. The first is to determine the arrays of local extremas, maxima, and minima from the input signal *x*(*t*). In the next step, based on the obtained results, two envelopes *e_up_*(*t*) and *e_dow_*(*t*) are built using cubic spline interpolation. The arithmetic mean of the envelope *m*_1_(*t*) is determined:

(1) $$m_{1}\left(t\right)=\frac{e_{up}\left(t\right)+e_{dow}\left(t\right)}{2},$$

The mean *m*_1_(*t*) is subtracted from the input signal *x*(*t*), giving *d*_1_(*t*):

(2) $$d_{1}\left(t\right)=x\left(t\right)-m_{1}\left(t\right),\;$$

The function *d*_1_(*t*), often called proto-IMF [85], is considered a component of IMF when it meets two conditions: the number of extremas *d*_1_(*t*) and the number of zero-crossings are equal or differ by at most one, and, at each point *d*_1_(*t*), the average of the local maxima and minima envelopes is zero. The received IMFs are frequency ordered components. The subtraction of *d*_1_(*t*) as IMF results in the procedure being repeated for the rest of the signal:

(3) $$h_{1}\left(t\right)=x\left(t\right)-d_{1}\left(t\right)\;$$

until the assumed stoppage criterion is reached. The signal remaining after the procedure is called the residual signal *r*(*t*).

The results obtained using the discussed classical EMD procedure, however, did not always provide satisfactory results due to the occurring phenomenon of leakage of frequency components between the IMFs. This phenomenon is known as the mode mixing problem [86,87,88]. In order to limit its impact on the obtained results, a slightly different approach was proposed, consisting in giving a noise-assisted signal to the input in each attempt, enriched with the component white noise *w_n_* [89]:

(4) $$y_{n}\left(t\right)=x\left(t\right)+w_{n}\left(t\right).\;$$

During the experiment, the EEMD of the standard deviation parameters of the added noise at 0.2 was used, and the number of ensemble trials was N = 100. The trend *r*(*t*), related to deviations and distortions caused by sensor drift, was removed in the study of VAG signals. The reduction in *r*(*t*) was the final stage of signal preprocessing. Each signal was normalized to an amplitude range between 0 and 1. The signal purified in this way was subjected to further tests, which are described in detail in the first part of the article.

### 2.6. Feature Extraction

The number of signal indices (parameters) used in vibroacoustic diagnostics is very large. The selection of the signal measures used in this paper was based on a literature analysis [90,91,92,93,94] and the previous experience of the authors presented in papers [51,52,95].

As with the femoral-tibial joint research described in Part I, 12 potentially useful markers in detecting degenerative knee lesions were analyzed. Those are:

- The mean value (MV) is one of the most popular intuitive descriptive statistics. It is the sum of the values of a measurable characteristic divided by the number of units of a finite statistical population.
  (5) $$\bar{x}=\frac{1}{N}\overset{N}{\underset{i=1}{\sum }}x_{i}$$
  where:
  $x_{i}$ is the value of the discrete signal at the nth point, *n* = 1, …, *N*;
  N is the number of samples in the signal.
- The straightened average value (SA) is the average value from the absolute value; the use of this parameter allows eliminating the phenomenon of the average value approaching zero, especially visible for oscillatory signals.
  (6) $$\bar{x}=\frac{1}{N}\overset{N}{\underset{i=1}{\sum }}\left|x_{i}\right|$$
- The root mean square (RMS) is defined as the square root of the mean square. This parameter is not sensitive to sudden changes manifested by single peaks in the signal.
  (7) $$x_{\mathrm{RMS}}=\sqrt{\frac{1}{N}\overset{N}{\underset{i=1}{\sum }}x_{i}{}^{2}}$$
- The peak value (PV), also called the maximum value of the signal, as opposed to RMS, is an indicator that is highly sensitive to rapid changes in the state of the test objects.
  (8) $$\hat{x}=max\left|x_{i}\right|$$
- The peak to peak value (PPV) is the amplitude measured from the largest top to the largest bottom of the wave, unlike PV, the two extremes, smallest and largest, are considered.
  (9) $$x_{\mathrm{PPV}}=\left|x_{max}-x_{min}\right|$$
- The crest factor (CF) is a measure that gives the ratio of the peak value (PV) to the RMS value of the signal.
  (10) $$x_{\mathrm{CF}}=\frac{\hat{x}}{x_{\mathrm{RMS}}}$$
- The impact factor (IF) is defined similarly to the CF, except that the denominator in this case is the mean value (MV). Its diagnostic properties are also similar to those of CF, but it is more sensitive.
  (11) $$x_{I}=\frac{\hat{x}}{\bar{x}}$$
- The shape factor (SF) is a measure giving the ratio of the RMS value to the mean of the absolute value (SA).
  (12) $$x_{\mathrm{SF}}=\frac{x_{\mathrm{RMS}}}{\bar{x}}$$
- The variance (VAR) is a measure of the dispersion of the sample results around the distribution center; it is the expected value of the square of the variance of a random variable minus its population mean.
  (13) $$x_{\mathrm{VAR}}^{2}=\frac{1}{N-1}\overset{N}{\underset{i=1}{\sum }}{\left(x_{i}-\bar{x}\right)}^{2}$$
- Kurtosis (KUR) is a measure that describes the degree of concentration of outcomes in a distribution. It is a fourth-order central moment. It provides information about the degree of similarity of the data scattered around the mean with respect to a normal distribution.
  (14) $$x_{\mathrm{KUR}}=\frac{\frac{1}{N}\sum_{i=1}^{N}{\left(x_{i}-\bar{x}\right)}^{4}}{{\left[\frac{1}{N}\sum_{i=1}^{N}{\left(x_{i}-\bar{x}\right)}^{2}\right]}^{2}}$$
- The M6A parameter is the sixth central moment normalized by the variance raised to the third power. This coefficient is more sensitive to the presence of pulses in the signal. It is defined as:
  (15) $$x_{M6A}=\frac{\frac{1}{N}\sum_{i=1}^{N}{\left(x_{i}-\bar{x}\right)}^{6}}{{\left[\frac{1}{N}\sum_{i=1}^{N}{\left(x_{i}-\bar{x}\right)}^{2}\right]}^{3}}$$
- The M8A parameter is known as the eighth central moment, normalized by the variance to the fourth power. It is defined as:
  (16) $$x_{M8A}=\frac{\frac{1}{N}\sum_{i=1}^{N}{\left(x_{i}-\bar{x}\right)}^{8}}{{\left[\frac{1}{N}\sum_{i=1}^{N}{\left(x_{i}-\bar{x}\right)}^{2}\right]}^{4}}$$

A detailed description of the indicators is presented in Part I of this paper and earlier papers by the authors [51,52,95,96].

### 2.7. Selection of Optimal Signal Features

Due to the different locations of the mounted sensors and the different characteristics of the relative cartilage movement during the patellar tests, it was necessary to reduce the parameters under analysis. Such a procedure, as already confirmed in the previous studies presented in Part I, favors the increase in the generalizing abilities of the neuronal networks while reducing the costs of calculations. OKC movement occurs in one primary axis and is characterized by a rotary stress pattern in the joint; whereas, CKC is characterized by a linear stress pattern, and joint motion occurs in multiple axes. Moreover, in OKC only one segment of the joint is moving in comparison to CKC in which simultaneous movement occurs in both knee segments. Therefore, VAG was performed in both closed and open kinetic chains in order to address fully the complex biomechanics of the patellofemoral joint.

There are many methods for selecting optimal signal features. Commonly used methods include techniques such as chi-square [97,98], tree-based feature selection [99,100], Pearson’s correlation [101,102], LASSO [103,104,105], low variance [106], and recursive feature elimination [107].

The Neighborhood Component Analysis (NCA) Algorithm was used to discard redundant features in the analyzed issue. Optimal signal measures were selected separately for each of the analyzed variants. NCA is a nonparametric method of selecting features in order to maximize the accuracy of the regression and classification algorithms. The discussed machine learning technique is used to classify multidimensional data into specific groups, in accordance with the given distance metric. It is based on looking for a linear transformation of the input data, so that the average classification efficiency without a single output is maximized. NCA is a method developed on the basis of the Nearest Neighbor (KNN) algorithm [108,109].

Let us assume a specific training set as:

(17) $$\;S=\left\{\left(x_{i},y_{i}\right),\;i=1,2,\ldots ,N\right\},$$

where *x_i_* described *d*-dimensional vector, *y_i_* ∈{1,2,…,c} is its corresponding label, *c* is the number of classes, and N is an observations number. The main task then is to determine the weight vector *w*, allowing the selection of the nearest neighbor properties, which optimizes the classification. The weighted distance between the samples *x_i_* and *x_j_* is shown in the equation [108,109]:

(18) $$D_{w}\left(x_{i},x_{j}\right)=\overset{d}{\underset{l=1}{\sum }}w_{l}^{2}\left|x_{il}-x_{jl}\right|,$$

where *w_l_* is a weight associated with the *l-*th feature. It is necessary to determine the probability of the correct classification *p_ij_* [108,109]:

(19) $$pij=\{\begin{array}{c} 0,\;\;\mathrm{if}\;i=j\\ \frac{\kappa \left(D_{w}\left(x_{i},x_{j}\right)\right)}{\sum_{k\neq i}\kappa \left(D_{w}\left(x_{i},x_{k}\right)\right)},\;\mathrm{if}\;i\neq j \end{array}\;.$$

The relationship between *p_ij_* and the derived distance *D_w_* is defined using the kernel function *κ*, defined as:

(20) $$\;\kappa \left(z\right)=\exp\left(-\frac{z}{\sigma }\right),$$

where *σ* denotes the kernel width, which is the input parameter that affects the probability of selecting each point as a reference point. The probability of correct classification could be defined as [108,109]:

(21) $$\;p_{i}=\overset{N}{\underset{j-1,j\neq i}{\sum }}y_{ij}p_{ij},$$

where

(22) $$y_{ij}=\{\begin{array}{c} 1,\;\;\;\mathrm{if}\;y_{i}=y_{j}\\ 0,\;\mathrm{otherwise} \end{array}$$

As mentioned earlier, one of the assumptions of NCA is to obtain the highest possible classification efficiency by omitting one of the inputs. The approximate accuracy could be written as [108,109]:

(23) $$F\left(w\right)=\overset{N}{\underset{i=1}{\sum }}p_{i}.$$

In order to maximize accuracy, the regularized objective term *λ* is introduced. Then, *F(w)* can be written as [108,109]:

(24) $$F\left(w\right)=\overset{N}{\underset{i=1}{\sum }}p_{i}-\lambda \;\overset{d}{\underset{l=1}{\sum }}w_{l}{}^{2}.$$

For the best results, term *λ* is tuned via cross validation. The next step is to find the derivative with respect to the gradient *w_l_* [108,109]:

(25) $$\;\frac{\partial F\left(w\right)}{\partial w_{l}}=2\left(\frac{1}{\sigma }\underset{i=1}{\sum }\left(p_{i}\underset{k\neq i}{\sum }p_{ij}\left|x_{il}-x_{jl}\right|-\underset{j=1}{\sum }y_{ij}p_{ij}\left|x_{il}-x_{jl}\right|\right)-\lambda \right)w_{l}.$$

The minimal classification loss is obtained for the best *λ* selection.

### 2.8. Artificial Neural Networks

Artificial neural networks (ANN) are one type of highly parameterized statistical models. They are capable of mapping complex functions in a way similar to the operation of brains of living organisms [110,111]. One of the most popular applications of neural networks is solving classification problems. The network in this application is a tool that allows assigning the studied objects to different classes [112,113].

Among the different types of neural networks, one of the most popular is the multilayer perceptron (MLP). It is characterized by a layered arrangement of neurons and unidirectional data flow (from input to output) without feedback [114,115]. The training of MLP-type networks is possible by using the backward error propagation method. The input signals are multiplied by coefficients called synaptic weights and then summed. The excitation level, thus determined, becomes the argument of a transition function (activation function) that calculates the output of the neuron.

A special variety of artificial neural networks are radial basis function (RBF) networks, in which the hidden neuron implements a function that varies radially around a selected center. These are unidirectional three-layer networks consisting of an input layer, a hidden layer, and an output layer. The input neurons pass data to the hidden neurons, as in MLP type networks. In the hidden layer, there are radial basis functions, which are equivalent to the hidden neurons [116,117].

Artificial Neural Networks with radial basis functions (RBF) and multilayer perceptron (MLP) networks were used as a tool for case classification in the problem under consideration [114,115].

The Statistica 13.1 package (Tulsa, OK, USA) containing modules including machine learning and artificial neural networks was used for the calculations. Three classification variants were considered in the analyzed problem. In each of them, in addition to the selected features of acoustic signals recorded by a sensor located on the patella, parameters such as age, sex, and BMI (Body Mass Index) were also considered as inputs. Variant I included analysis of indices recorded in closed kinetic chain (sit to stand movement), variant II included indices recorded in open kinetic chain (limb straightening and bending in free hanging), while variant III included analysis of indices in both kinetic chains. Different sets of subdivisions of data into teaching, validation, and testing sets, among others, 50-25-25 and 60-20-20 were tested; the results are presented for the subdivision in which data were randomly divided into 70% for teaching, 15% for testing, and 15% for validation. At the output, a simplified classification system for articular cartilage damage was proposed. The classification involved assigning a set of features to one of two classes: 1. healthy cartilage and 2. cartilage for further diagnosis and surgical treatment (grade I to IV damage according to the ICRS scale).

## 3. Results and Discussion

### 3.1. Selection of Optimal Signal Features

The results of the selection of optimal signal measures for each of the three considered variants, obtained using the neighborhood component analysis (NCA) algorithm, are presented below.

**Figure 6:**
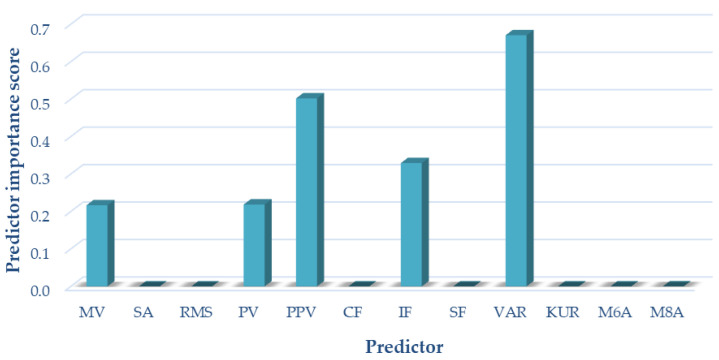
Selection of optimal features for variant I (OKC).

For variant I (sensor located on the patella, motion in an open kinetic chain), the following measures of acoustic signals were selected based on the analyses performed using the NCA algorithm: mean value, peak value, interpeak value, impulsivity coefficient, and variance. These measures were used as input data in variant I of the classification. The results of selecting the optimal measures for variant I are shown in Figure 6.

**Figure 7:**
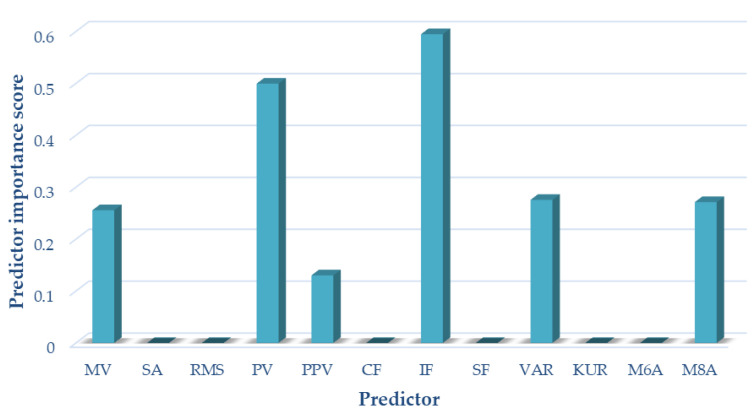
Selection of optimal features for variant II (CKC).

For variant II (sensor located on the patella, motion in a closed kinetic chain), the following measures of acoustic signals were selected based on the analyses performed using the NCA algorithm: mean value, peak value, interpeak value, impulsivity coefficient, variance, and M8A. These measures were used as input data for the variant II classification. The results of selecting the optimal measures for variant II are shown in Figure 7.

**Figure 8:**
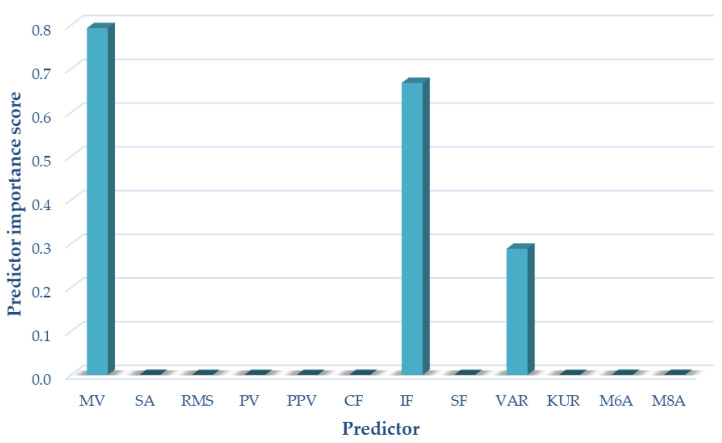
Selection of optimal features for variant III (OKC and CKC).

For variant III (sensor located on the patella, motion in a closed and open kinetic chain), the following measures of acoustic signals were selected on the basis of the analyses performed using the NCA algorithm: mean value impulsivity coefficient and variance. These measures were used as input data in variant III of the classification. The results of the selection of optimal measures for variant III are shown in Figure 8.

### 3.2. Classification

The results for the most accurate classifiers proposed by the automatic neural network selection algorithm of the Statistica package for one case each of multilayer perceptron (MLP) and radial basis function (RBF) networks, respectively, are presented below. The detailed results of the learning, testing, and validation accuracy for each network in all the analyzed variants are presented in Table 2. The accuracy of the network is given separately for the learning and test data. The accuracy measure used depended on the type of output variable. For continuous variables (regression), it was the correlation coefficient, calculated for learning, testing, and validation data (if such data were used). For qualitative variables (classification), the relative number of cases correctly classified (relative to the total number of cases) is reported.

The data describing the network type and structure are shown in Table 2. The numerical notation following the network type describes the number of neurons in the input layer, the number of neurons in the hidden layer, and the number of network outputs, respectively. The next three columns report the accuracy of the network, separately for the learning and test data. The highest accuracy of learning (98.53%) was obtained for the MLP network in Variant I and the RBF network in Variant II, while the lowest (91.91%) for the RBF network in Variant III, which included the analysis of both kinetic chains. In the case of testing accuracy, the highest value (100.00%) was observed for the MLP network in all analyzed variants and the lowest value for the RBF network in variant I. The highest value (100.00%) was observed for the RBF network in variants I and II as well as MLP network in variant I. The lowest value was observed for MLP network in variant I. Detailed information on the parameters such as the learning algorithm, error function, and activation functions for individual networks for all variants considered are presented in Table 2. The detailed results for classification in each group (HC and OA) and overall classification accuracy for all three variants are presented in Table 3.

The highest classification accuracy in the HC group of 98.88% of correctly assigned cases was obtained for the MLP network in variant III, while the lowest accuracy was obtained for the RBF network in the same variant. In the case of the OA group, the highest accuracy of 100% of cases correctly assigned to the classes was obtained for the MLP network in variant II as well as for the RBF network in variants I and II, while the lowest (78.26%) was for the MLP network in variant I. The highest total classification accuracy in both groups was achieved by the RBF networks for variant I and MLP for variant II; it was 98.53% of correctly assigned cases. The RBF network with the best classification performance had nine input neurons (nine input variables), thirty-five neurons in the hidden layer, and two output neurons (assigning data to one of the two classes HC and OA). The learning algorithm used was RBFT, the error function was Entropy, the activation function in the hidden layer was the Gaussian function, and in the output layer, it was Softmax. The MLP network with the best classification performance had ten input neurons (ten input variables), sixteen neurons in the hidden layer, and two output neurons. The learning algorithm used was BFGS 17, the error function was Entropy, the activation function in the hidden layer was a logistic function, and in the output layer, it was Softmax. The lowest accuracy was obtained for the MLP network in variant I, with 89.71% correctly assigned to each class of cases.

**Figure 9:**
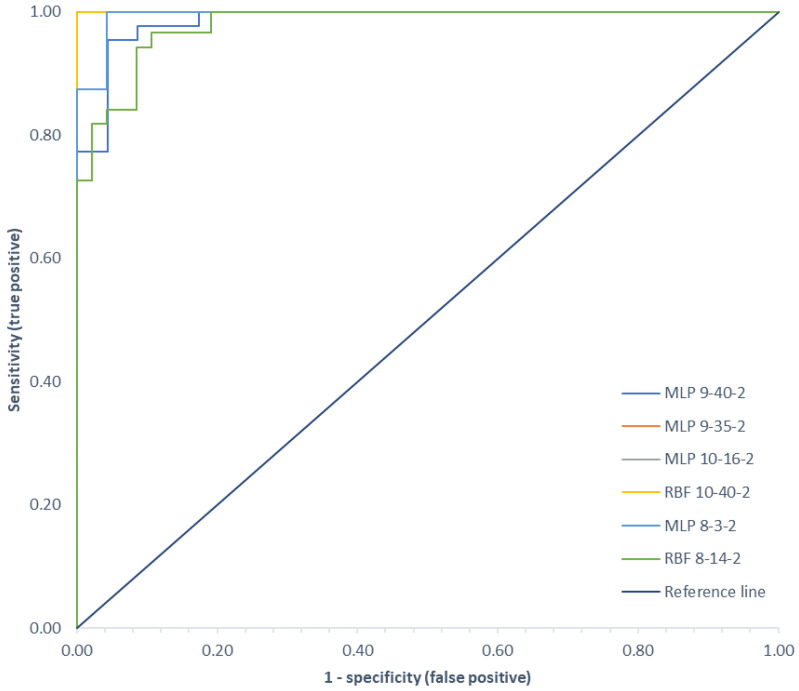
Comparison of ROC curves for all classification variants.

A summary of the Receiver Operating Characteristic (ROC) curves for each classifier for all variants analyzed is shown in Figure 9. Information on the area under the curves and the threshold values for each curve are summarized in Table 4.

ROC curves are a graphical representation of the effectiveness of a predictive model by plotting the qualitative characteristics of the binary classifiers produced from the model using multiple different cutoff points. They are widely used in medicine to evaluate the diagnostic accuracy of a test, to select the optimal test cutoff point, and to compare the diagnostic accuracy of several tests [22,118]. ROC curves are also used in evaluating techniques for machine learning. Classifiers that draw curves closer to the upper left corner show better performance. As a reference point, a random classifier is expected to produce points that are located along the diagonal. The closer the curve approaches the 45-degree diagonal of the ROC plot space, the less accurate the test. The largest area under the ROC curve of 1.00 was observed for the MLP network in variant II and the RBF network in variants I and II, and the smallest area (0.979) was for the RBF network in variant III.

The accuracy of the classification demonstrates the good performance of this proposed noninvasive diagnosis of articular cartilage injuries of the patellofemoral joint. The results also indicate that MLP as well as RBF type networks performed well in solving the studied problem. The results show that, similar to the diagnosis of femoral-tibial joint cartilage damage, there was no improvement in classification quality with increased data from two kinetic chains. In the case of the patellofemoral joint, very similar results were obtained for variant I and II analyses. Based on these, it can be concluded that it is optimal to use the test protocol for a single kinetic chain, and the reduced amount of data, in this case, did not adversely affect the obtained results of calibration. This will shorten the study and reduce the amount of data analyzed without compromising the quality of classification.

Those findings corresponded with our previous study, in which the evaluation provided excellent diagnostic accuracy reaching 96.32% for the tibiofemoral joint and one kinetic chain. However, for the tibiofemoral joint the OKC showed higher, but not statistically significant, diagnostic accuracy in comparison to the patellofemoral joint, where CKC showed the highest AUC reaching 1.00. This finding shows that for suspected OA in one compartment of the knee joint one kinetic chain is sufficient; however, the combination of VAG performed in both kinetic chains provided the best overall evaluation of the knee joint. A comparison of the diagnostic results for different anatomic locations for all evaluation variants analyzed is shown in Table 5.

The analysis of the obtained classification parameters shows that in addition to the kinetic chain, the placement of sensors can affect the quality of the obtained classification results. The best classification accuracy for all considered variants was obtained for the patellofemoral joint at 98.53%, with a sensitivity of 0.958, and a specificity of 1.000. It was obtained by the RBF network in variant I and the MLP network in variant II. In the case of the femoral-tibial joint, the best classification performance was obtained for the MLP network in variant I, where signals recorded in the open kinetic chain were analyzed. The classification accuracy in this case was 96.32%, with a sensitivity of 0.957, and a specificity of 0.967.

The F1-score is a measure of model precision on a dataset and is most commonly used to evaluate binary classification systems. It is a way of combining model precision and recall and is defined as the harmonic mean of model precision and recall [119]. The highest F1 score values of 0.979 were obtained for the patellofemoral joint for analyses in Variant I and II. For the femoropopliteal joint, the highest value of 0.946 was obtained for the MLP in Variant I.

The Matthews correlation coefficient (MCC) is a measure of the quality of binary (two-class) classifications, introduced by Brian W. Matthews [120]. The Matthews correlation coefficient is one of the most informative single scores to determine the prediction quality of a binary classifier in the context of a confusion matrix [121]. It can be used even in cases where the two classes have very different sizes. The highest MCC value of 0.968 was obtained for the patellofemoral joint for single kinetic chains for the RBF-type network in variant I and for the MLP in variant II. For the patellofemoral joint, the highest value of 0.918 was obtained for the MLP in OKC.

The results observed are consistent with the results obtained for the sensitivity and F1 score. The difference in the results may be related to the level of vibration attenuation by the skin (less influence in the case of the patella) and the amount of artifacts caused by the muscles moving in the direct vicinity of the sensor (more influence in the case of registration on the lateral and medial side at the level of the joint fissure). The classification accuracy was slightly better for analyses in variants I and II, where motion was analyzed in single kinetic chains for both the femoral-tibial and patellofemoral joints. The lack of improvement in classification quality with the increased amount of data (two kinetic chains) indicates that the testing protocol can be reduced to single kinetic chain analyses, and the choice of chain is dependent on the sensor location.

Our findings also correspond with other authors in regard to diagnostic accuracy where sensitivity ranged from 0.56 to 1, depending on sensors used and the signal processing algorithms [60,61,122]. A comparison of the diagnostic results of the proposed method with those reported in related works is summarized in Table 6. The data in this table are arranged in ascending order of classification accuracy.

As shown in Table 6, there has been a wide spread of results obtained by researchers. Krishnan et al. [60] showed the lowest accuracy among all cited papers; however, during this study, the researchers did not concentrate on the cartilage itself and evaluated all intra-articular pathologies including ligament and menisci tears. Moreover, there was no information about severity of the lesions, which could also affect the sensitivity of the method in this study. Further, the classification method in this study was the simplest, which could affect the overall results. RBF and MLP proved to be the most accurate in evaluating cartilage status as presented in our studies and Rangayyan et al. [61]. Interestingly, the same authors, based on the same classification and study group, showed different results where diagnostic accuracy ranged from 77% [55] to 100% [61]. This shows that the classification accuracy is strongly related to the signal preprocessing methods and the set of signal measures selected used on the classifier inputs. Our proposed method not only shows high sensitivity and specificity for cartilage lesions but also is clinically applicable due to the ease of the testing protocol. Patients are free to perform the test in their comfort zone, while the range of motion required for testing is 0–90, which can be performed by most patients with cartilage lesions. Moreover, movement time does not impede our results, which was an issue in previous studies, where a set timeline of the study was proposed for testing [61,129,132]. Selecting the best signal processing algorithm and examination protocol is still a question to be answered in the future. Multiple different approaches have been proposed in the literature [49,133], and further studies are required to establish a future gold standard for VAG in knee evaluation.

### 3.3. Limitations of the Study and Future Plans

The limitation of this study is the use of a simplified classification model including the assignment of results to only two groups: healthy cartilage and damaged cartilage, without differentiating the degree of damage. The authors plan to conduct a study focused on precise determination of the degree of cartilage damage by assigning the results to one class of degree of damage according to the ICRS scale; however, a larger study group is required to achieve this goal. Another potential limitation of this study is that the TKR group patients presented high grade diffused cartilage lesions, for which sensitivity seems be higher than for focal low grade lesions. Further limitations of the study also undoubtedly include the variation in age between groups, and further studies need to be conducted in groups with less variation. The study focused only on cartilage surface damage; whereas in clinical practice, simultaneous damage to many different structures of the knee joint is usually encountered.

Planned research will include expansion of the study group, extension of algorithms for signal preprocessing, feature extraction, and methods for selecting optimal signal measures. The authors plan to test various feature extraction methods for vibroacoustic signals such as chi-square, tree-based feature selection, Pearson’s correlation, LASSO, low variance, and recursive feature elimination. The authors also plan to test various classification methods based on machine learning and deep learning methods. In future work, the results of comparative analyses will be presented to determine the most important vibroacoustic signal measures and optimal classification methods to create a test protocol that can be used in the clinical evaluation of damage to cartilage and other structures of the knee joint. The planned end result is to propose a simple testing protocol and assessment of joint condition based on recorded vibroacoustic signals, possible to perform in any orthopedic practice.

## 4. Conclusions

In response to the increasing public concern about knee osteoarthritis, this paper proposes a method for processing acoustic signals and selecting optimal signal measures using the NCA algorithm. A classification accuracy of >95% was obtained for most of the analyzed variants. The obtained results confirmed the thesis that inexpensive, noninvasive, and, most importantly, effective diagnosis of damage to articular cartilage covering the articular surfaces of the patellofemoral joint based on generated vibroacoustic signals is possible. This confirms the validity of the assumptions made and the usefulness of the proposed method created based on statistical parameters and machine learning. Application of the NCA algorithm allowed for a reduction in the amount of input data, improvement in the quality of classification, and reduction in the computation time. For the patellofemoral joint, the best classification performance was obtained for separate analysis of signals recorded for the open (OKC) and closed (CKC) kinetic chains. This suggests a simplification of the testing protocol to limb movement in a single kinetic chain. Simplification of the testing protocol and reduction in the requirements proposed in our study can ease clinical application of the method in general practitioners’ and orthopedic clinics.

## Figures and Tables

**Table 1:** Characteristics of the study participants.

Study Group	N	Males/ Females	Age (years ± SD)	Height (m ± SD)	Weight (kg ± SD)	BMI	Tegner-Lyshom Score
Healthy control (HC)	33	9/24	24.10 ± 5.56	1.71 ± 0.09	65.16 ± 15.10	21.95 ± 3.09	100 ± 0.0
Osteoarthritis (OA)	34	15/19	56.15 ± 12.99	1.69 ± 0.09	89.08 ± 14.30	31.19 ± 4.83	38.59 ± 12.96

**Table 2:** Accuracy of the MLP and RBF neural network for variant I (open kinetic chain), II (closed kinetic chain), and III (open and closed kinetic chain).

Variant	Network Name	Accuracy of Learning (%)	Accuracy of Testing (%)	Accuracy of Validation (%)	Learning Algorithm	Error Function	Activation (Hidden)	Activation (Output)
I	MLP 9-40-2	89.71	100.00	85.71	BFGS 25	SOS	Linear	Exponential
	RBF 9-35-2	98.53	85.71	100.00	RBFT	Entropy	Gauss	Softmax
II	MLP 10-16-2	98.53	100.00	100.00	BFGS 17	Entropy	Logistic	Softmax
	RBF 10-40-2	97.06	92.86	100.00	RBFT	Entropy	Gauss	Softmax
III	MLP 8-3-2	97.79	100.00	96.43	BFGS 103	Entropy	Tanh	Softmax
	RBF 8-14-2	91.91	96.43	96.43	RBFT	Entropy	Gauss	Softmax

**Table 3:** Summary of the classification accuracy of the MLP and RBF networks for variant I, II and III.

Network Name		HC	OA	Total
MLP 9-40-2	Total	45.00	23.00	68.00
	Correct	43.00	18.00	61.00
	Correct (%)	95.56	78.26	89.71
RBF 9-35-2	Total	45.00	23.00	68.00
	Correct	44.00	23.00	67.00
	Correct (%)	97.78	100.00	98.53
MLP 10-16-2	Total	45.00	23.00	68.00
	Correct	44.00	23.00	67.00
	Correct (%)	97.78	100.00	98.53
RBF 10-40-2	Total	45.00	23.00	68.00
	Correct	43.00	23.00	66.00
	Correct (%)	95.56	100.00	97.06
MLP 8-3-2	Total	89.00	47.00	136.00
	Correct	88.00	45.00	133.00
	Correct (%)	98.88	95.74	97.79
RBF 8-14-2	Total	89.00	47.00	136.00
	Correct	85.00	40.00	125.00
	Correct (%)	95.51	85.11	91.91

**Table 4:** Area under the ROC curves and ROC threshold.

	Variant I		Variant II		Variant III	
	MPL	RBF	MPL	RBF	MPL	RBF
ROC area	0.986	1.000	1.000	1.000	0.995	0.979
ROC Threshold	0.533	0.480	0.470	0.437	0.866	0.620

**Table 5:** Comparison of diagnostic results for different anatomical locations for all variants.

Location	Variant	Network Name	Accuracy (%)	Sensitivity	Specificity	AUC	Precision	Recall	F1 Score	MCC
Femoral-Tibial Joint	I	MLP 13-9-2	96.32	0.957	0.967	0.996	0.936	0.957	0.946	0.918
		RBF 13-43-2	89.71	0.867	0.912	0.960	0.830	0.867	0.848	0.771
	II	MLP 15-12-2	94.85	0.935	0.956	0.989	0.915	0.935	0.925	0.886
		RBF 15-6-2	91.91	0.950	0.906	0.977	0.809	0.950	0.874	0.820
	III	MLP 15-24-2	93.70	0.928	0.941	0.977	0.875	0.928	0.901	0.855
		RBF 15-5-2	89.63	0.806	0.948	0.974	0.898	0.806	0.849	0.773
Patellofemoral joint	I	MLP 9-40-2	89.71	0.900	0.896	0.986	0.783	0.900	0.837	0.766
		RBF 9-35-2	98.53	0.958	1.000	1.000	1.000	0.958	0.979	0.968
	II	MLP 10-16-2	98.53	0.958	1.000	1.000	1.000	0.958	0.979	0.968
		RBF 10-40-2	97.06	0.920	1.000	1.000	1.000	0.920	0.958	0.938
	III	MLP 8-3-2	97.79	0.978	0.978	0.995	0.957	0.978	0.968	0.951
		RBF 8-14-2	91.91	0.909	0.924	0.979	0.851	0.909	0.879	0.819

**Table 6:** Comparison diagnostic results of proposed method with other related works.

Authors	Classification Methods	Accuracy (%)	Sensitivity	Specificity	AUC
Krishnan et al. [60]	Logistic regression analysis	68.90	0.564	0.784	N/A
Umpathy and Krishnan [122]	Linear discriminant analysis	76.40	0.789	0.745	N/A
Rangayyan and Wu [55]	RBF	77.53	0.711	0.824	0.832
Mascarenhas et al. [123]	Random forest	80.89	0.868	0.765	0.817
Sharma and Acharya [124]	LS-SVM	89.89	0.914	0.889	N/A
Wu and Krishnan [125]	Multiple classifier Fusion system	80.9	0.895	0.922	0.948
Rangayyan and Wu [126]	RBF	82.02	0.711	0.902	0.820
Nalband et al. [127]	LS-SVM	83.14	0.981	0.622	0.671
Wu et al. 45	Bayesian decision rule	86.67	0.750	0.936	0.910
Shidore et al. [128]	SVM	87.69	0.857	0.838	0.926
Yang et al. [129]	Bayesian decision rule	88.00	0.714	0.979	0.957
Cai et al. [53]	Dynamic weighted classifier Fusion system	88.76	0.737	1.000	0.952
Rangayyan and Wu [130]	RBF	89.89	0.921	0.882	0.917
Mu et al. [131]	Strict 2-surface proximal classifier	91.01	0.947	0.882	0.950
Kim et al. [132]	Back propagation neural network	95.4	0.920	0.987	N/A
Karpiński et al. [96]	MLP, RBF	96.32	0.957	0.967	0.996
Proposed method	MLP, RBF	98.53	0.958	1.000	1.000
Rangayyan et al. [61]	RBF	100	1	1	0.961

## Data Availability

Data presented in this study are available from the corresponding authors upon request.
